# Frequency of metastases within the hypothalamic–pituitary area and the associated high-risk factors in patients with brain metastases

**DOI:** 10.3389/fneur.2023.1285662

**Published:** 2023-11-30

**Authors:** Peng Xie, Huiling Hu, Xiong Cao, Ning Lan, Huanyu Zhang, Ruifeng Yan, Peng Yue, Wenteng Hu, Hui Qiao

**Affiliations:** ^1^Department of Radiation Oncology, The First Hospital of Lanzhou University, Lanzhou, China; ^2^CT Room, The First People’s Hospital of Lanzhou City, Lanzhou, China; ^3^Department of Thoracic Surgery, The First Hospital of Lanzhou University, Lanzhou, China; ^4^Department of Radiotherapy, The First Affiliated Hospital of Xi’an Jiaotong University, Xi’an, China; ^5^Department of Radiology, The First Hospital of Lanzhou University, Lanzhou, China; ^6^Department of Oncology, The First Hospital of Lanzhou University, Lanzhou, China; ^7^School of Life Sciences, Lanzhou University, Lanzhou, China

**Keywords:** hypothalamus, pituitary, brain metastasis, frequency, whole brain radiotherapy

## Abstract

**Objective:**

Brain radiotherapy often results in impairment of hypothalamic–pituitary (HT-P) function, which in turn causes secretory dysfunction of related hormones. In this paper, the frequency of metastasis in the HT-P area and its high-risk factors in patients with brain metastasis were retrospectively analyzed, and thus provide experimental evidence for protecting HT-P area during whole brain radiotherapy (WBRT).

**Methods:**

A retrospective analysis was performed on the data of patients with brain metastasis diagnosed by cranial magnetic resonance imaging (MRI) at the First Hospital of Lanzhou University from 2017 to 2020. The anatomical positions of the hypothalamus and pituitary were delineated, followed by their expansion by 5 mm outwards, respectively, in the three-dimensional direction, and the hypothalamus +5 mm and pituitary +5 mm were obtained as the avoidance area, in which the frequency of brain metastasis was evaluated. Univariate and multivariate logistic regression models were used to analyze the high risk factors of brain metastasis in HT-P area.

**Results:**

A total of 3,375 brain metastatic lesions from 411 patients were included in the analysis. The rates of brain metastasis in the hypothalamus +5 mm and pituitary +5 mm in the whole group of cases were 2.9% (12/411) and 1.5% (6/411) respectively; the frequency of lesions was 0.4% (13/3375) and 0.2% (6/3375) respectively. Univariate and multivariate analyses showed that the number of brain metastases (OR = 14.946; 95% CI = 4.071–54.880; *p* < 0.001), and the occurrence of brain metastasis in the pituitary (OR = 13.331; 95% CI = 1.511–117.620; *p* = 0.020) were related to brain metastasis in the hypothalamus, and that the only relevant factor for brain metastasis in the pituitary was the occurrence of that in the hypothalamus (OR = 0.069; 95% CI = 0.010–0.461; *p* = 0.006). There was no correlation between tumor pathological types, the maximum diameter, the total volume of brain metastatic lesions and the risk of brain metastasis in hypothalamus and pituitary.

**Conclusion:**

The frequency of brain metastasis in the HT-P area is extremely low. The risk of brain metastases in the hypothalamus is correlated with their number. The larger the number of metastatic lesions, the higher the frequency of brain metastasis. Protection of the HT-P area during WBRT may be unlikely to compromise the tumor recurrence rate for patients with a relatively small number of brain metastases.

## Introduction

It has been reported in studies that up to 40% of tumor patients will develop brain metastasis (1). Radiotherapy is an important therapy for brain metastasis, and whole brain radiotherapy (WBRT) is a commonly used treatment modality, especially for patients with a large number of brain metastases (2, 3). Prophylactic WBRT is an important component in the treatment of small-cell lung cancer (4). With the advancement of medical treatment and radiotherapy technology, the survival time of patients with brain metastasis has been greatly prolonged, and people have began to pay more attention to the toxicities and side effects as well as quality of life which are related with treatment. WBRT can cause neurocognitive dysfunction, which is related to hippocampal injury caused by radiotherapy (5, 6). Several studies have shown that hippocampal protection during WBRT can reduce the risk of cognitive impairment (7, 8). WBRT technologies for hippocampal protection have been widely applied in clinic. However, in addition to neurocognitive dysfunction caused by hippocampal damage, radiotherapy-induced hypothalamic–pituitary (HT-P) dysfunction is also a concern that is not uncommonly seen in clinic (9–11). In the past, more attention was paid to HT-P dysfunction caused by radiotherapy for head and neck or brain tumors in clinic (12–14). WBRT-induced HT-P dysfunction is often overlooked due to its low dose irradiation. However, more and more studies have suggested that receiving low-dose irradiation of 18-40Gy in the HT-P area can also cause secretory dysfunction of related hormones (9, 10, 15, 16). Therefore, it needs to be considered whether or not to protect this area during WBRT, which, however, depends on the frequency of brain metastasis in it. Unfortunately, there was few research reports on the frequency of brain metastasis in the HT-P area in the past, and there was also a lack of scientific evidence in this regard. Therefore, it is necessary to investigate the frequency of brain metastasis in the HT-P area and associated risk factors. In this study, we retrospectively analyse the data of patients with brain metastasis, and thus provide experimental evidence for protecting HT-P area during WBRT.

## Methods

### Patient selection

The data on 411 patients with brain metastasis admitted to the First Hospital of Lanzhou University from 2017 to 2020 were retrospectively collected. The screening criteria for them were as follows: eligibility criteria (malignant tumors diagnosed by pathology and brain metastasis diagnosed by T1/ T2-weighted plain scan sequences of cranial magnetic resonance imaging (MRI), T1-weighted enhanced sequences, etc.) and exclusion criteria (a medical history of more than 2 types of malignant tumors, combined with other craniocerebral diseases, or incomplete data).

### Localization of brain metastasis and methods for delineating the hypothalamus and pituitary

**Figure 1:**
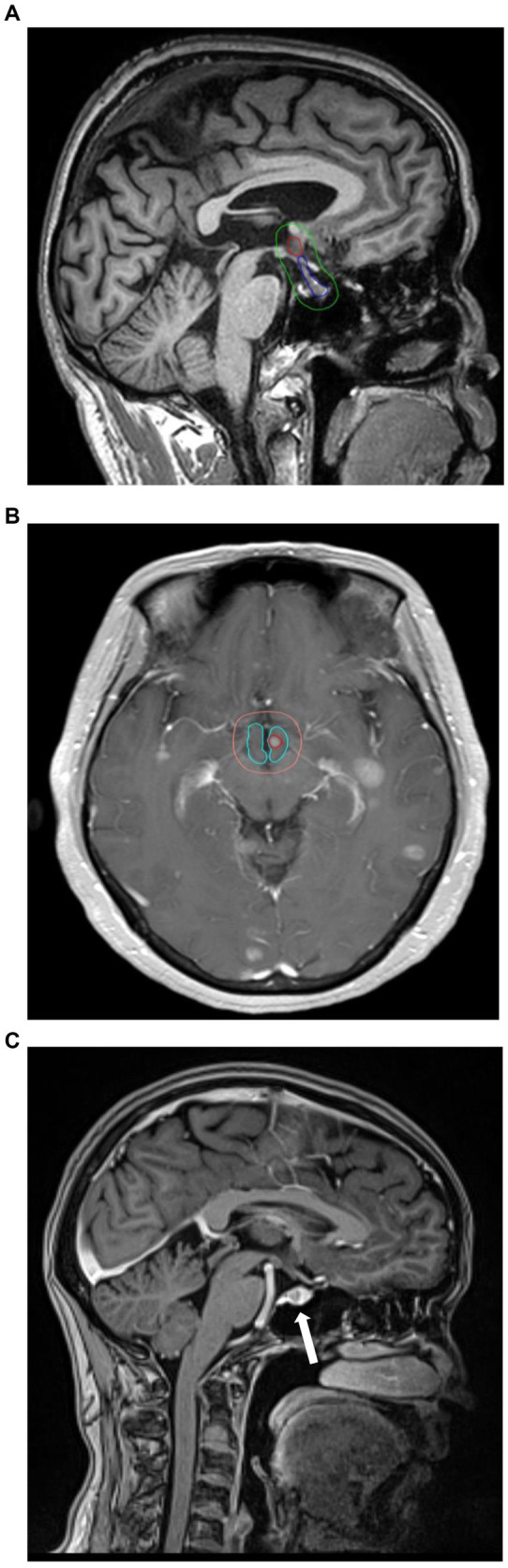
**(A)** The red line range is the location of the hypothalamus, the blue line range is the location of the pituitary, and the green line range is the hypothalamic–pituitary (+5 mm). area. **(B)** Example for hypothalamic metastasis. The red circles are metastases. **(C)** Example for pituitary metastasis. The arrow points to the metastasis.

The cranial MRI images of patients at initial diagnosis were accessed and viewed through the hospital PACS system. The number and distribution of brain metastases were observed on T1, T2 weighted plain scanned and T1 weighted enhanced images. The MRI T1-weighted enhanced sequence images were imported into Varian eclipse planning system for target delineation to delineate the anatomical boundaries of all brain metastasis, hypothalamus and pituitary, which were expanded by 5 mm outwards in the three-dimensional direction to obtain the boundary contours of hypothalamus +5 mm and pituitary +5 mm, respectively. The boundary distance of brain metastatic lesions and the shortest distance between the boundaries of the two areas was measured. The schematic diagram of the location of brain metastatic lesions, hypothalamus and pituitary is shown in Figure. 1.

### Research content

The frequency of brain metastasis was calculated in the hypothalamus +5 mm and pituitary +5 mm. The relationship between brain metastasis in the hypothalamus and pituitary and various clinical features was analyzed by statistical methods, and the high-risk factors for brain metastasis in this area were explored.

### Statistical methods

Logistic regression method was adopted to analyze the correlation between brain metastasis in HT-P area and clinical factors such as age, sex, pathological type, as well as the number, the maximum diameter and the total volume of brain metastatic lesions by SPSS 21 software. The optimal cut-off value for the number of brain metastases was obtained by calculating the area under the receiver operating characteristic (AUROC) curve. The difference was statistically significant (*p* < 0.05).

## Results

A total of 425 patients with brain metastasis were screened at our institution from 2017 to 2020. Among them: 3 patients had more than two tumor diseases at the same time, 11 patients did not have complete medical records. Specifically, we do not have a detailed record of how they were diagnosed and treated. Finally, a total of 3,375 brain metastatic lesions from 411 patients were included in the analysis. There were 249 males and 162 females. The age of the patients in the whole group ranged from 18 to 86 years, with a median age of 62 years. The median of brain metastatic lesions was 3 in patients of the whole group, and the cases with ≥10 and ≥ 20 lesions accounted for 22.9% (94/411) and about 10.0% (41/411), respectively. There were 341 cases (83.0%) of lung cancer, 30 cases (7.3%) of breast cancer, and 40 cases (9.7%) of other tumors (Table 1). Per patient, the rate of brain metastasis occuring in the hypothalamus+5 mm was 2.9% (12/411); per brain metastasis, the rate of hypothalamus lesions was 0.4% (13/3375). Per patient, the rate of brain metastasis occuring in the pituitary +5 mm was 1.5% (6/411); per brain metastasis, the rate of pituitary lesions was 0.2% (6/3375). Univariate and multivariate analyses showed that the number of brain metastases (OR = 14.946; 95% CI = 4.071–54.880; *p* < 0.001) and the occurrence of brain metastasis in the pituitary (OR = 13.331; 95% CI = 1.511–117.620; *p* = 0.020) were correlated with the risk of brain metastasis in the hypothalamus (Table 2). The results of univariate and multivariate analyses of brain metastasis in the pituitary indicated that brain metastasis in the hypothalamus is the only risk factor for that in the pituitary (OR = 0.069; 95% CI = 0.010–0.461; *p* = 0.006) (Table 3). There was no correlation between sex, age, tumor pathological types, the maximum diameter, the total volume of brain metastatic lesions and the risk of brain metastasis occurring in hypothalamus and pituitary.

**Table 1:** Clinical characteristics of 411 patients diagnosed with BMs.

Parameters	Numbers of patients	Percentage (%)
Sex		
Male	249	60.6
Female	162	39.4
Age in years		
<65	247	60.1
≥65	164	39.9
Median		62
Range		18–86
Primary tumours		
Lung cancer	341	83.0
Non-small cell lung cancer	236	57.4
Small cell lung cancer	105	25.5
Breast cancer	30	7.3
Others	40	9.7
Number of BMs Mean: 8.2, median: 3 (range: 1–126)		
Maximum diameter of BMs (mm) Mean: 16.6, median: 13 (range: 2 to 58)		
Aggregate volume of BMs(mm^3^) Mean: 11368.2, median: 2610 (range: 24 to 148,005)		

**Table 2:** Logistic regression analysis for frequency of metastases within 5 mm of the hypothalamus.

	Univariate regression analysis			Multivariate logistic regression analysis		
	OR	95%CI	value of *p*	OR	95%CI	value of *p*
Sex, *n* (%)						
Male 249 (60.6%)	1					
Female 162 (39.4%)	1.247	0.374–4.159	0.719			
Age, *n* (%)						
<65 years 247 (60.1%)	1					
≥65 years164 (39.9%)	0.884	0.254–3.071	0.846			
Pathology, *n* (%)						
1 NSCLC 236 (57.4%)	1					
2 SCLC 105 (25.5%)	0.973	0.246–3.844	0.969			
3 Breast 30 (7.3%)	1.074	0.128–9.044	0.948			
4 Others 40 (9.7%)	0	—	0.998			
Number of BM, *n* (%)						
<25,380 (92.5%)	1					
≥25 31 (7.5%)	17.136	4.889–60.061	0.000*	14.946	4.071–54.880	0.000*
Maximal diameter of BM (mm), *n* (%)						
<13,202 (49.1%)	1					
≥13,209 (50.9%)	0.869	0.261–2.896	0.819			
Volume of BM (mm^3^), *n* (%)						
<2,610 204 (49.6%)	1					
≥2,610 207 (50.4%)	1.844	0.531–6.403	0.335			
BM in pituitary, *n* (%)						
No 405 (98.5%)	1					
Yes 6 (1.5%)	21	3.397–129.812	0.001*	13.331	1.511–117.620	0.020*

**Table 3:** Logistic regression analysis for frequency of metastases within 5 mm of the pituitary gland.

	Univariate regression analysis			Multivariate logistic regression analysis		
	OR	95%CI	value of *p*	OR	95%CI	value of *p*
Sex, *n* (%)						
Male 249 (60.6%)	1					
Female 162 (39.4%)	0.802	0.240–2.673	0.719			
Age, *n* (%)						
<65 years 247 (60.1%)	1					
≥65 years 164 (39.9%)	0.773	0.140–4.272	0.768			
Pathology, *n* (%)						
1 NSCLC 236 (57.4%)	1					
2 SCLC 105 (25.5%)	3.484	0.573–21.187	0.175			
3 Breast 30 (7.3%)	3.845	0.338–43.736	0.278			
4 Others 40 (9.7%)	0	—	0.998			
Number of BM, *n* (%)						
<8,290(70.6%)	1					
≥8,121(29.4%)	4.911	0.887–27.193	0.068	0.283	0.047–1.688	0.166
Maximal diameter of BM (mm), *n* (%)						
<13,202 (49.1%)	1					
≥13,209 (50.9%)	1.048	0.209–5.255	0.955			
Volume of BM (mm^3^), *n* (%)						
<2,610 204 (49.6%)	1					
≥2,610 207 (50.4%)	5.293	0.613–45.721	0.13			
BM in hypothalamus, *n* (%)						
No 399 (97.1%)	1					
Yes 12 (2.9%)	21	3.397–129.812	0.001*	0.069	0.010–0.461	0.006*

## Discussion

It has been reported in many studies that the probability of functional impairment of endocrine organs such as hypothalamus and pituitary after radiotherapy can be as high as 20–90% (10, 11, 17, 18). The side effects caused by impaired function of these organs usually include decreased secretion of these hormones such growth and adrenocorticotropic hormone (19, 20), fatigue and sexual dysfunction, etc. (21, 22). However, in the past, more attention was paid to dysfunction in the HT-P area caused by radiotherapy for head and neck or brain tumors, with no importance attached to the damage to this area after WBRT in patients with brain metastasis. The author of this paper believed that there are two main reasons for this. First, dose-fractionation patterns of WBRT are usually 37.5Gy/15f, 30Gy/10f, 40Gy/20f, etc. (23, 24), with relatively low radiation dose, which is lower than the conventional limiting dose for pituitary in clinic (Dmax<50Gy) (25). In fact, for some radiotherapy units, there is even a higher limiting dose to pituitary, such as Dmax<60Gy (16). However, for the hypothalamus, no radiation dose limitation is often imposed on it. In the report on quantitative analysis of normal tissue effects in the clinic (QUANTEC) published in 2010, there was also no recommendation for the radiation dose limitation to organs such as hypothalamus and pituitary (26). Radiologists believe that relatively low radiation dose can not cause functional damage to such organs as hypothalamus and pituitary. Therefore, in delineating the target area of WBRT, the HT-P area is generally not deliberately protected, and also the radiation dose to these two organs is not routinely evaluated. Second, in the past, the survival time of patients with brain metastasis was short, usually only a few months. The mid- and long-term toxic and side effects on these patients were not taken serious after craniocerebral radiotherapy. However, thanks to the progress in medical drugs and radiotherapy technology, the median survival time of patients with brain metastasis from lung cancer with EGFR mutation has reached 25 months (27). It has also been found in studies that HT-P dysfunction caused by brain radiotherapy may occur as early as 3–6 months after treatment (10, 11). Today, when patients with brain metastasis generally achieve long-term survival, the damage to HT-P function caused by WBRT deserves more and more attention.

Similar to the problem of hippocampal protection, if the frequency of brain metastasis in HT-P area is extremely low, the possibility of tumor recurrence in this area will be relatively low. Therefore, this area can be protected during WBRT. In other words, when the location of brain metastatic lesions in patients is relatively far from the HT-P area, the avoidance of these two areas can be considered in delineating the target area for WBRT, or stricter dose restrictions can be imposed on these two organs in evaluating the radiotherapy plan, thus making them receiving as low a radiation dose as possible. Therefore, there is a need to understand the frequency of brain metastasis in the HT-P area. However, there were few reports on the frequency of brain metastasis in HT-P area in the past, with the scarcity of relevant data. Therefore, the data on patients with brain metastasis at our unit were retrospectively analyzed.

In 2010, Marsh analyzed a total of 935 brain metastasis in 155 patients in a study, and found only one case of brain metastasis in the pituitary (28). A larger sample was reported from a retrospective study by Janssen (29). In this study, a total of 4,280 brain metastatic lesions in 865 patients were included in the analysis. The results showed that the hypothalamic area was involved in only 26 patients (3%, 26/865), and the pituitary area was involved in 9 ones (1%, 9/865). It has also been reported in literature that the rate of pituitary metastasis in autopsy of cancer patients is only 1.9% (30). These results all indicated that the rate of brain metastasis in HT-P area is extremely low. The results of this study showed that the brain metastasis rates in the hypothalamus +5 mm and pituitary +5 mm were 2.9% (12/411) and 1.5% (6/411) respectively, and that the frequency of lesions was 0.4% (13/3375) and 0.2% (6/3375) respectively, indicating that the brain metastasis rate in HT-P area is extremely low, which is similar to the results reported in previous studies (29). Univariate and multivariate analyses showed that the number of brain metastases was correlated with the risk of brain metastasis in the hypothalamus. When the number of brain metastases ≥25, the risk of brain metastasis in the hypothalamus increased nearly 15 times. In other words, the larger the number of brain metastases, the higher the risk of brain metastasis in the hypothalamus.

However, similar to Janssen’s findings in 2019 (29), our study also showed no correlation between the probability of brain metastasis in the pituitary and the number of brain metastases. Additionally, the results of our study showed that tumor pathological types, the maximum diameter and the total volume of brain metastatic lesions were not correlated with the risk of brain metastasis in the hypothalamus and pituitary. In this study, the frequencies of metastases occurring in the hypothalmus and pituitary appear to be interrelated. Univariate and multivariate analyses suggest that the risk of pituitary metastasis increased in patients with brain metastases in the hypothalamus, and similarly that risk of hypothalamic metastases was greater in patients with pituitary metastases. This result was not observed in previous studies. However, we acknowledge the small numbers of positive cases of brain metastasis in the hypothalamus and pituitary in our study which may limit interpretation of these results. Among 411 patients with brain metastasis in our study, there were only 6 cases of pituitary metastasis and 12 cases of hypothalamic metastasis. However, it was found that there were 2 patients with brain metastasis in both hypothalamus and pituitary, which may affect the statistical results. It still needs to be further verified by the studies with more and larger samples whether the risk of brain metastasis in the hypothalamus is correlated with that in the pituitary area.

In the retrieval of the literature, it was found that there were almost no reports on the frequency of brain metastasis in the HT-P area in Chinese population. As a data analysis of a large sample of patients with brain metastasis, this study provides a scientific basis for investigating the risk of brain metastasis in the HT-P area in Chinese population, with a certain reference value. Also, it has been pointed out in studies that it is feasible to protect the HT-P area during WBRT with the help of current advanced radiotherapy technology (31, 32). If this is taken into account, this study will appear to be more meaningful. However, as a retrospective analysis, the study had certain limitations. First, there was inevitable selection bias in case collection. Second, cranial MRI images of all patients were from a single point in time, and does not evaluate the risk of cerebral metastases arising in the HP-axis over time. Third, there were as many as 10 histological types in the whole group of patients in this study. Except lung cancer, the number of cases of other pathological types was small, which may fail to better reflect the risk of brain metastasis in the HT-P area in non lung cancer patients.

## Conclusion

In conclusion, the frequency of brain metastasis in the HT-P area is extremely low. There is a correlation between the number of brain metastases and the risk of brain metastasis in the hypothalamus. In other words, the larger the number of metastatic lesions, the higher the frequency of brain metastasis in the hypothalamus. It needs to be further verified by more studies whether there is a correlation between the risk of brain metastasis in the hypothalamus and that in the pituitary. Therefore, for patients with a relatively small number of brain metastases, this data suggests that protection of the HT-P area during WBRT may be unlikely to compromise the tumor recurrence rate; clinical applications should be explored.

## Data availability statement

The raw data supporting the conclusions of this article will be made available by the authors, without undue reservation.

## Ethics statement

Ethical review and approval was not required for the study on human participants in accordance with the local legislation and institutional requirements. Written informed consent from the patients/participants or patients/participants legal guardian/next of kin was not required to participate in this study in accordance with the national legislation and the institutional requirements.

## Author contributions

PX: Writing – original draft. HH: Investigation, Writing – review & editing. XC: Investigation, Writing – review & editing. NL: Data curation, Formal analysis, Writing – review & editing. HZ: Data curation, Writing – review & editing. RY: Investigation, Writing – review & editing. PY: Data curation, Investigation, Writing – review & editing. WH: Data curation, Investigation, Writing – review & editing. HQ: Writing – review & editing.

## References

[1] Khuntia D Brown P Li J Mehta MP. Whole-brain radiotherapy in the management of brain metastasis. J Clin Oncol. (2006) 24:1295–304. doi: 10.1200/JCO.2005.04.6185 16525185

[2] Oehlke O Wucherpfennig D Fels F Frings L Egger K Weyerbrock A. Whole brain irradiation with hippocampal sparing and dose escalation on multiple brain metastases: local tumour control and survival. Strahlenther Onkol. (2015) 191:461–9. doi: 10.1007/s00066-014-0808-9, PMID: 25592907

[3] Mulvenna P Nankivell M Barton R Faivre-Finn C Wilson P McColl E. Dexamethasone and supportive care with or without whole brain radiotherapy in treating patients with non-small cell lung cancer with brain metastases unsuitable for resection or stereotactic radiotherapy (QUARTZ): results from a phase 3, non-inferiority, randomised trial. Lancet. (2016) 388:2004–14. doi: 10.1016/S0140-6736(16)30825-X, PMID: 27604504 PMC5082599

[4] Pechoux CL Sun A Slotman BJ Ruysscher DD Belderbos J Gore EM. Prophylactic cranial irradiation for patients with lung cancer. Lancet Oncol. (2016) 17:e277–93. doi: 10.1016/S1470-2045(16)30065-1 27396646

[5] Abayomi OK. Pathogenesis of irradiation-induced cognitive (lysfunction). ActaOnc-ol. (1996) 35:659–63. 10.3109/02841869609083995 8938210

[6] Gondi V Tome WA Mehta MP. Why avoid the hippocampus? A comprehensive review. Radiother Oncol. (2010) 97:370–6. doi: 10.1016/j.radonc.2010.09.013, PMID: 20970214 PMC2997490

[7] Gondi V Pugh SL Tome WA Caine C Corn B Kanner A. Preservation of memory with conformal avoidance of the hippocampal neural stem-cell compartment during whole-brain radiotherapy for brain metastases (RTOG0933): a phase II multi-institutional trial. J Clin Oncol. (2014) 32:3810–6. doi: 10.1200/JCO.2014.57.2909 25349290 PMC4239303

[8] Brown PD Gondi V Pugh S Tome WA Wefel JS Armstrong TS. Hippocampal Avoi-dance during whole-brain radiotherapy plus Memantine for patients with brain metastases: phase III trial NRG oncology CC001. J Clin Oncol. (2020) 38:1019–29. doi: 10.1200/JCO.19.02767, PMID: 32058845 PMC7106984

[9] Darzy KH. Radiation-induced hypopituitarism after cancer therapy: who, how and when to test. Nat Clin Pract Endocrinol Metab. (2009) 5:88–99. doi: 10.1038/ncpendmet1051, PMID: 19165221

[10] Mehta P Fahlbusch FB Rades D Schmid SM Gebauer J Janssen S. Are hypothalamic- pituitary (HP) axis deficiencies after whole brain radiotherapy (WBRT) of relevance for adult cancer patients? - a systematic review of the literature. BMC Cancer. (2019) 19:1213. doi: 10.1186/s12885-019-6431-5, PMID: 31830931 PMC6909600

[11] Gebauer J Mehta P Fahlbusch FB Schmid SM Rades D Janssen S. Hypothalamic-pituitary Axis dysfunction after whole brain radiotherapy – a cohort study. Anticancer Res. (2020) 40:5787–92. doi: 10.21873/anticanres.14595, PMID: 32988906

[12] Kyriakakis N Lynch J Orme SM Gerrard G Hatfield P Loughrey C. Pituitary dysfunction following cranial radiotherapy for adult-onset nonpituitary brain tumours. Clin Endocrinol. (2016) 84:372–9. doi: 10.1111/cen.12969, PMID: 26501843

[13] Elson A Bovi J Kaur K Maas D Sinson G Schultz C. Effect of treatment modality on the hypothalamic-pituitary function of patients treated with radiation therapy for pituitary adenomas: hypothalamic dose and endocrine outcomes. Front Oncol. (2014) 4:73. doi: 10.3389/fonc.2014.00073 24782984 PMC3988389

[14] Ipekci SH Cakir M Kiyici A Koc O Artac M. Radiotherapy-induced hypopituitarism in nasopharyngeal carcinoma: the tip of an iceberg. Exp Clin Endocrinol Diabetes. (2015) 123:411–8. doi: 10.1055/s-0035-1549963, PMID: 26011172

[15] Appelman-Dijkstra NM Malgo F Neelis KJ Coremans I Biermasz NR Pereira AM. Pituitary dysfunction in adult patients after cranial irradiation for head and nasopharyngeal tumours. Radiother Oncol. (2014) 113:102–7. doi: 10.1016/j.radonc.2014.08.018, PMID: 25236713

[16] Scoccianti S Detti B Gadda D Greto D Furfaro I Meacci F. Organs at risk in the brain and their dose-constraints in adults and in children: a radiation oncologist’s guide for delineation in everyday practice. Radiother Oncol. (2015) 114:230–8. doi: 10.1016/j.radonc.2015.01.016, PMID: 25701297

[17] Darzy KH. Radiation-induced hypopituitarism. Curr Opin Endocrinol Diabetes Obes. (2013) 20:342–53. doi: 10.1097/MED.0b013e3283631820 23807607

[18] Kyriakakis N Lynch J Orme SM Gerrard G Hatfield P Short SC. Hypothalamic-pituitary axis irradiation dose thresholds for thedevelopment of hypopituitarism in adult-onset gliomas. Clin Endocrinol. (2019) 91:131–40. doi: 10.1111/cen.13971 30873631

[19] Madaschi S Fiorino C Losa M Lanzi R Mazza E Motta M. Time course of hypothalamic-pituitary deficiency in adults receiving cranial radiotherapy for primary extrasellar brain tumors. Radiother Oncol. (2011) 99:23–8. doi: 10.1016/j.radonc.2011.02.015, PMID: 21458091

[20] Velickovic N Drakulic D Petrovic S. Time-course of hypothalamic-pituitary-adrenal axis activity and inflammation in juvenile rat brain after cranial irradiation. Cell Mol Neurobiol. (2012) 32:1175–85. doi: 10.1007/s10571-012-9843-1, PMID: 22527859 PMC11498391

[21] Sinha A Hollingsworth KG Ball S Cheetham T. Impaired quality of life in growth hormone-deficient adults is independent of the altered skeletal muscle oxidative metabolism found in conditions with peripheral fatigue. Clin Endocrinol. (2014) 80:107–14. doi: 10.1111/cen.12252, PMID: 23711232

[22] Gebauer J Fick EM Waldmann A Langer T Kreitschmann-Andermahr I Lehnert H. Selfreported endocrine late effects in adults treated for brain tumours, Hodgkin and non-Hodgkin lymphoma: a registry-based study in northern Germany. Eur J Endocrinol. (2015) 173:139–48. doi: 10.1530/EJE-15-0174, PMID: 25947143

[23] Brown PD Ballman KV Cerhan JH Anderson SK Carrero XW Whitton AC. Postoperative stereotactic radiosurgery compared with whole brain radiotherapy for resected metastatic brain disease (NCCTG N107C/CEC·3): a multicentre, randomised, controlled, phase 3 trial. Lancet Oncol. (2017) 18:1049–60. doi: 10.1016/S1470-2045(17)30441-2, PMID: 28687377 PMC5568757

[24] Rades D Bohlen G Dunst J Lohynska R Veninga T Stalpers L. Comparison of short-course versus long-course whole-brain radiotherapy in the treatment of brain metastases. Strahlenther Onkol. (2008) 184:30–5. doi: 10.1007/s00066-008-1795-5, PMID: 18188520

[25] Pai HH Thornton A Katznelson L Finkelstein DM Adams JA Fullerton BC. Hypothalamic/pituitary function following high-dose conformal radiotherapy to the base of skull: demonstration of a dose–effect relationship using dose–volume histogram analysis. Int J Radiat Oncol Biol Phys. (2001) 49:1079–92. doi: 10.1016/S0360-3016(00)01387-0, PMID: 11240250

[26] Bentzen SM Constine LS Deasy JO Eisbruch A Jackson A Marks LB. Quantitative analyses of normal tissue effects in the clinic (QUANTEC): an introduction to the scientific issues. Int J Radiat Oncol Biol Phys. (2010) 76:S3–9. doi: 10.1016/j.ijrobp.2009.09.040 20171515 PMC3431964

[27] Magnuson WJ Lester-Coll NH Wu AJ Yang TJ Lockney NA Gerber NK. Management of Brain Metastases in tyrosine kinase inhibitor–Naïve epidermal growth factor receptor–mutant non–small-cell lung Cancer: a retrospective multi-institutional analysis. J Clin Oncol. (2017) 35:1070–7. doi: 10.1200/JCO.2016.69.7144, PMID: 28113019

[28] Marsh JC Garg S Wendt JA Gielda BT Turian JV Herskovic AM. Intracranial metastatic disease rarely involves the pituitary: retrospective analysis of 935 metastases in 155 patients and review of the literature. Pituitary. (2010) 13:260–5. doi: 10.1007/s11102-010-0229-4, PMID: 20405323

[29] Janssen S Mehta P Bartscht T Schmid SM Fahlbusch FB Rades D. Prevalence of metastases within the hypothalamic-pituitary area in patients with brain metastases. Radiat Oncol. (2019) 14:152. doi: 10.1186/s13014-019-1337-6, PMID: 31455428 PMC6712746

[30] He W Chen F Dalm B Kirby PA Greenlee JD. Metastatic involvement of the pituitary gland: a systematic review with pooled individual patient data analysis. Pituitary. (2015) 18:159–68. doi: 10.1007/s11102-014-0552-2, PMID: 24445565

[31] Mehta P Janssen S Fahlbusch FB Schmid SM Gebauer J Cremers F. Sparing the hippocampus and the hypothalamic- pituitary region during whole brain radiotherapy: a volumetric modulated arc therapy planning study. BMC Cancer. (2020) 20:610. doi: 10.1186/s12885-020-07091-x, PMID: 32605648 PMC7325372

[32] Fan XW Wang JQ Wu JL Wang HB Wu KL. Simultaneously avoiding the hippocampus and hypothalamic-pituitary axis during whole brain radiotherapy: a planning study. Med Dosim. (2019) 44:130–5. doi: 10.1016/j.meddos.2018.04.004, PMID: 29778320

